# Mast cell proliferation in the cerebrospinal fluid after intraventricular administration of anti-B7H3 immunotherapy

**DOI:** 10.1007/s00262-020-02824-0

**Published:** 2021-02-03

**Authors:** Kim Kramer, Maria A. Donzelli, Melissa S. Pessin

**Affiliations:** 1 Department of Pediatrics, Memorial Sloan Kettering Cancer Center, 1275 York Avenue, New York, NY 10065 USA; 2 Laboratory Medicine, Memorial Sloan Kettering Cancer Center, 1275 York Avenue, New York, NY 10065 USA

**Keywords:** Radioimmunotherapy, Omburtamab, Mastocytosis, Allergic reaction

## Abstract

Omburtamab is a B7H3-specific murine monoclonal antibody. B7H3 (CD 276) is a member of the B7 family of immune checkpoint co-inhibitory receptors overexpressed on many human malignancies. Radioimmunotherapy with ^124^I- or ^131^I-omburtamab administered in the cerebrospinal fluid (CSF), intraperitoneal or intratumoral cavity is currently under investigation for the treatment of CNS malignancies. The immunologic effects of anti-B7H3 therapy are not fully elucidated. A 6-year-old male was diagnosed with metastates of neuroblastoma to the received intraventricular ^131^I-omburtamab on an IRB-approved protocol. A treatment cycle consisted of a 2 mCi dosimetry dose and a 50 mCi treatment dose. Dosimetry by serial imaging, pharmacokinetics and safety were investigated. Clinical status, magnetic resonance imaging, CSF cell count and cytology were evaluated pre- and post-^131^I-omburtamab at 5 and 26 weeks. The patient did well with cycle 1. Three hours after the dosimetry dose of cycle 2, he developed a fever (39 °C), chills and headache. Blood and CSF samples were sent for culture. CSF was notable for nucleated cell pleocytosis with profound mast cell proliferation consistent with chemical meningitis. He was treated with supportive care; symptoms resolved over 48 h. Further therapy with ^131^I-omburtamab was electively discontinued. CSF cell count 5 weeks later demonstrated resolution of CSF pleocytosis. Local–regional administration of intraventricular ^131^I-omburtamab targeting B7H3 can result in a profound nucleated CSF pleocytosis with mastocytosis consistent with an acute allergic reaction.

## Introduction

B7H3 (CD 276) is a member of the B7 family of immune checkpoint co-inhibitory receptors playing a role in local T cell regulation. B7H3 functions beyond immune costimulation have been identified, with recent work indicating a crucial role for B7H3 in promoting carcinogenesis and metastases. (1) Overexpression of B7H3 protein has been demonstrated in many human malignancies (2) and is generally considered to be a negative prognostic marker. (3–5) Omburtamab (8H9) is a murine monoclonal antibody specific for B7H3. (2) Anti-B7H3 tumor-targeted radioimmunotherapy has been studied using ^124^I- or ^131^I-omburtamab administered in the cerebrospinal fluid (CSF), (6) intraperitoneum (7) and intratumor cavity. (8) The immunologic effects of anti-B7H3 therapy have not been fully studied.

## Case presentation

A 4-year-old male was diagnosed with stage 4 neuroblastoma involving a right adrenal mass, bones and bone marrow. Tumor biopsy showed amplification of the MYCN gene with unfavorable histology. He achieved a complete remission following standard multiagent chemotherapy, tandem high-dose myeloablative therapy with stem cell rescue and immunotherapy with anti-GD2 antibodies, GM-CSF and IL-2. Two years after initial diagnosis, a routine surveillance scan demonstrated a left temporal lobe brain tumor with subacute hemorrhage. Surgical resection confirmed an isolated central nervous system progression of neuroblastoma.

The patient underwent treatment according to Memorial Sloan Kettering Cancer Center salvage regimen consisting of craniospinal radiation therapy, chemotherapy and intraventricular ^131^I-omburtamab on a phase 2 IRB-approved protocol (NCT00089245) [1]. A treatment cycle consisted of a 2 mCi dosimetry dose and a 50 mCi treatment dose. Dosimetry was by serial imaging, pharmacokinetics by CSF and blood sampling and safety investigated during a 5-week period. CSF sampling for cell count, differential and cytology was routinely performed the day of omburtamab injections (weekly for 2 weeks) and then 5 weeks later at follow-up. Response and survival were noted by the clinical status and magnetic resonance imaging and CSF cytology pre- and then post-^131^I-omburtamab at 5 and 26 weeks.

**Fig. 1:**
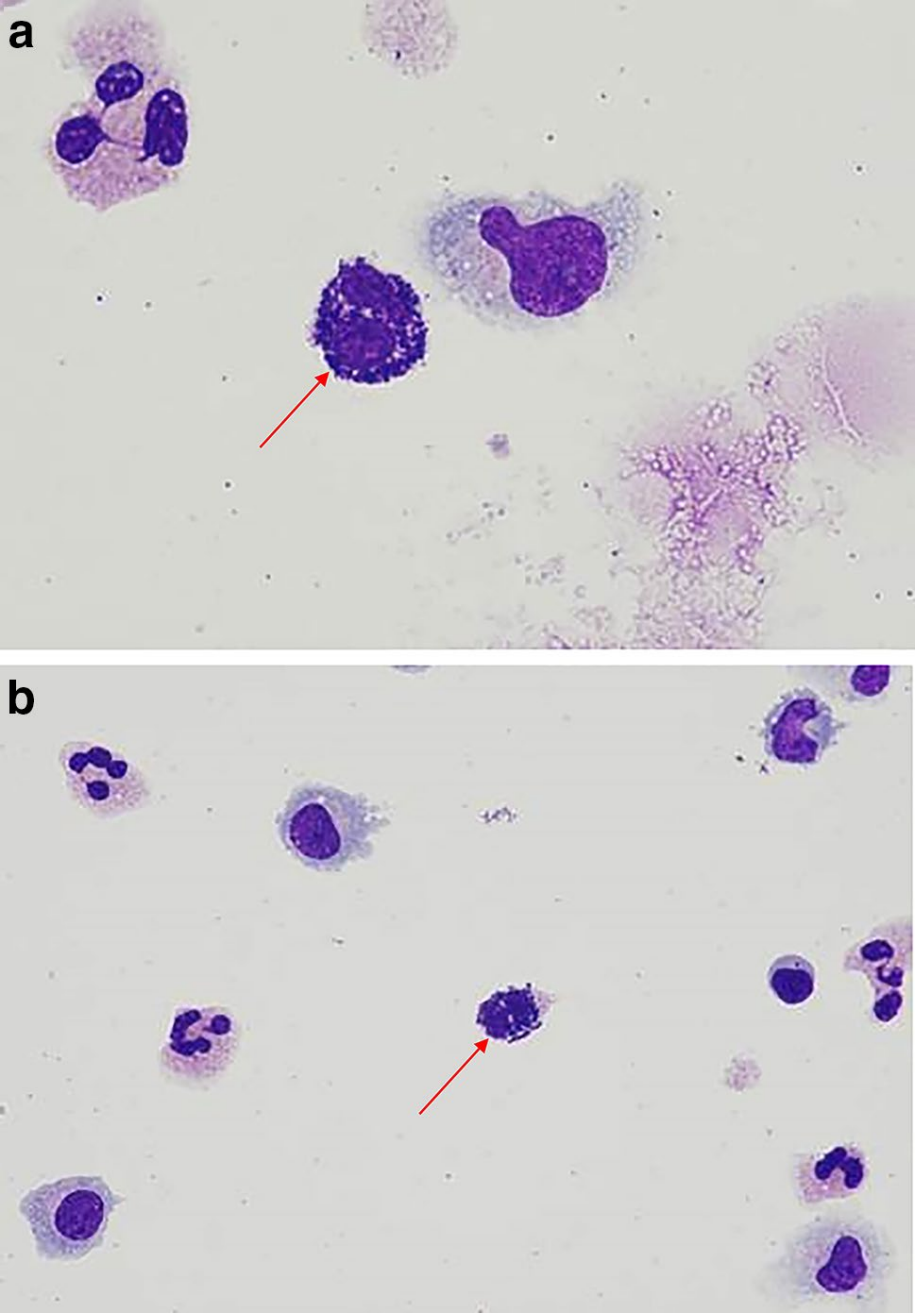
Patient CSF showing mastocytosis. **a**, **b** Abundant mastocytes adjacent to monocytes and lymphocytes in the CSF containing histamine and proteoglycan granules consistent with acute chemical meningitis following anti-B7H3 therapy

At baseline pre-^131^I-omburtamab, the patient was in a radiographic and cytologic remission. CSF was unremarkable for the presence of nucleated or extrinsic cells); total protein and glucose were within normal parameters. The patient did well with the first cycle consisting of the dosimetry and therapy dose. Five weeks after the completion of cycle 1, CSF on routine examination was notable for cell differential showing 66% lymphocytes, 33% monocytes and 1% eosinophils. Serum complete blood count was also unremarkable, total WBC 2.3 K/mcL, differential 67% neutrophils, 12.3% monocytes, 4.8% eosinophils and 14.9% lymphocytes. One week later, the patient proceeded to cycle 2. Three hours following the dosimetry dose of the second cycle (2 mCi ^131^I-omburtamab), the patient developed a high fever (39 °C) with chills and headache, approximately 3 h after administration. Blood and CSF samples were sent for culture. Serum complete blood count was unremarkable (WBC 2.9 K/mcL with differential 73% neutrophils, 16.4% monocytes and 9.2% lymphocytes). CSF was notable for nucleated cell pleocytosis (26 uL) initially interpreted as polymorphic white blood cells with abundant intracellular and extracellular bacteria. Further evaluation showed these cells were mast cells with basophilic granules consistent with mastocytosis (Fig. 1a, b), with few or no other atypical lymphocytes or monocytes. Blood and CSF cultures remained negative for bacterial growth. The patient was treated with supportive care including acetaminophen, dexamethasone and intravenous fluids for a mast cell induced chemical meningitis. Symptoms resolved over the next 48 h. Further therapy with ^131^I-omburtamab was electively discontinued. CSF cell count 5 weeks later demonstrated resolution of the CSF pleocytosis.

## Discussion

B7H3 (CD 276) is a member of the B7 family of immune checkpoint co-inhibitory receptors playing a role in local T cell regulation. B7H3 functions beyond immune costimulation have been identified, with recent work indicating a crucial role for B7H3 in promoting carcinogenesis and metastases [2]. Overexpression of B7H3 protein has been demonstrated in many human malignancies [3] and is generally considered to be a negative prognostic marker [4–6]. Omburtamab (8H9) is a murine monoclonal antibody specific for B7H3 [3]. Anti-B7H3 tumor-targeted radioimmunotherapy has been studied using ^124^I- or ^131^I-omburtamab administered in the cerebrospinal fluid (CSF) [1], intraperitoneum [7] and intratumor cavity [8]. The immunologic effects of anti-B7H3 therapy have not been fully studied.

As a co-stimulatory molecule of the cell surface B7 protein superfamily, B7H3 is a potentially powerful therapeutic target, whose overexpression on many human solid tumors has been demonstrated. Intraventricular administration of anti-B7H3-targeted therapy has been a therapeutic modality for many years, often resulting in a CSF pleocytosis with monocytes and lymphocytes. We note that among > 200 patients treated with nearly 600 injections of intraventricular ^131^I-omburtamab, atypical chemical meningitis as an adverse event has been noted rarely (< 10%) but that such reactions are typically associated with atypical lymphocytosis or macrophage proliferation. As a murine antibody, omburtamab might be expected to elicit an allergic reaction; however, this is the first instance whereby anti-B7H3 therapy resulted in a mastocytosis. Mast cells mediate inflammatory responses such as hypersensitivity and allergic reactions and as such are not routinely detected in the CSF. In our patient, the acute onset of fever and chills accompanied by headache appears to have been due to the chemical mediators in the mast cell degranulation process, producing the responses typical of an allergic reaction. For a minority of individuals predisposed to an allergic reaction, the previous exposure to murine anti-B7H3 therapy in cycle 1 may “prime” or sensitize them by stimulating the production of IgE antibodies, which attach to mast cells and basophils resulting in an allergic reaction. Future patients may benefit from serum or CSF IgE levels if the setting of mastocytosis. While this patient did not receive further therapy for concern of another drug-related adverse event, further analysis to learn more about mast cell recruitment in the CSF may be undertaken if a similar adverse event is observed in future patients.

Although B7H3-targeted therapy is designed to stimulate immune response against tumor cells, therapies that recruit inhibitory molecules like Fas ligand (Fas-L), transforming growth factor beta (TGF-β) and prostaglandin E2 (PGE2) may decrease the cytotoxicity by altering the immunosuppressive tumor microenvironment [9]. It is unclear how an influx of mast cells can alter the inhibitory receptors present in the tumor microenvironment and whether T cell metabolism and T cell signaling of the release of extracellular vesicles such as exosomes may change [10]. While the role of the tumor microenvironment and its effects on imposing barriers to metabolism and activity of tumor infiltrating lymphocytes is an area of active research, the immediate effects of cytotoxicity through beta-emission are hypothesized to be unaffected. However, the long-term effects of direct B7H3 inhibition through immune effector cells and the tumor microenvironment are less understood.

We now add the recruitment of mast cells into the CSF compartment to the immune phenomena exhibited when targeting B7H3. While targeting tumor-associated B7H3 as the next generation of immune checkpoint therapy is actively investigated in the form of monoclonal antibodies, small molecules and chimeric antigen receptor T cells, it is important to recognize the potential for occurrence of this phenomena. Local–regional administration of intraventricular ^131^I-omburtamab targeting B7-H3 can result in a profound nucleated CSF pleocytosis with mastocytosis consistent with an acute allergic reaction.

## Availability of data and material

Data for this case report are presented below. No additional data will be provided for this report.

## Data Availability

Not available.
