# Correction to: Mast cell proliferation in the cerebrospinal fluid after intraventricular administration of anti‑B7H3 immunotherapy

**DOI:** 10.1007/s00262-021-02942-3

**Published:** 2021-04-30

**Authors:** Kim Kramer, Maria A. Donzelli, Melissa S. Pessin

**Affiliations:** 1 Department of Pediatrics, Memorial Sloan Kettering Cancer Center, 1275 York Avenue, New York, NY 10065 USA; 2 Laboratory Medicine, Memorial Sloan Kettering Cancer Center, 1275 York Avenue, New York, NY 10065 USA

## Correction to: Cancer Immunology, Immunotherapy 10.1007/s00262-020-02824-0

The original version of this article unfortunately contained a mistake. NCT number is incorrect. The correct number is NCT03275402.

In section “Case presentation”, first sentence of the second paragraph should read as

The patient underwent treatment according to Memorial Sloan Kettering Cancer Center salvage regimen consisting of craniospinal radiation therapy, chemotherapy and intraventricular ^131^I-omburtamab on a phase 2 IRB-approved protocol (NCT03275402) [1].

Ethics approval section should read as

Patient (proxy) consented to be treated on IRB approved protocol registered to clinicaltrials.gov NCT03275402.

